# Superficial fungal infections in children in Northern Finland between 2000 and 2021: a single-center study

**DOI:** 10.1128/spectrum.01637-25

**Published:** 2025-08-28

**Authors:** Kiia Korpi, Eetu Kiviniemi, Jari Jokelainen, Laura Huilaja, Suvi-Päivikki Sinikumpu

**Affiliations:** 1 Faculty of Medicine, University of Oulu 6370, Oulu, Finland; 2 Northern Finland Birth Cohorts, Arctic Biobank, and Infrastructure for Population Studies, University of Oulu 6370, Oulu, Finland; 3 Department of Dermatology, Oulu University Hospital, Finland and Research Unit of Clinical Medicine, University of Oulu 6370, Oulu, Finland; MultiCare Health System, Tacoma, Washington, USA

**Keywords:** tinea, skin infection, tinea pedis, epidemiology

## Abstract

Superficial fungal infections in children are less prevalent compared with adults, and there are limited data on epidemiology in Northern Europe. This study retrospectively observed the epidemiological factors and treatment patterns of these infections in children in Northern Finland between 2000 and 2021. Patients under 18 years diagnosed with superficial tinea infections at the dermatology outpatient clinic of Oulu University Hospital were included in the study. During the study period, there were 94 cases fulfilling the inclusion criteria. The most common infections were tinea unguium (44.7%), tinea capitis (25.5%), and tinea pedis (23.4%). *Trichophyton rubrum* was the predominant pathogen (56.4%) in these infections. The duration of symptoms before referral to the dermatology clinic was over 6 months in most of the cases (53.2%). In this study, we found that superficial fungal infections are relatively rare in children and seem to have a delay in diagnosis. Increased awareness among physicians is important for accurate diagnosis and treatment.

**IMPORTANCE:**

Superficial fungal infections are infections of the skin and nails caused by fungi. They occur in 20%–25% of the global population. These infections are less common in children than in adults. This study investigated the prevalence of superficial fungal infections diagnosed in a Finnish hospital. The data were collected from patients diagnosed with superficial fungal infection at the age of under 18 years by a dermatologist between the years 2000 and 2021. Fungal infections of nails were the most common. Additionally, the study found frequent delays in diagnosis, as more than half of the cases had had symptoms for more than 6 months. This research provides information on fungal infections in children in Northern Finland and highlights the importance of improved recognition of these infections.

## INTRODUCTION

Superficial fungal infections are a prevalent global health concern, affecting an estimated 20%–25% of the population worldwide (1). The incidence of superficial fungal infections varies geographically and is influenced by socioeconomic conditions, climate, healthcare infrastructure, and individuals’ immune status (1). Typically, these infections are categorized based on their location (1, 2). Tinea capitis and tinea corporis are the most common superficial fungal infections in children, and their prevalence is higher in children than in adults. Tinea capitis occurs principally in the pediatric population (1, 3).

Superficial fungal infections are caused by three genera of dermatophytes: *Trichophyton (T.), Microsporum (M*.), and *Epidermophyton (E*.) (1, 3). The diagnosis of superficial fungal infections is recommended to be confirmed with a fungal culture or nucleic acid amplification test (NAAT) (4, 5), as the clinical manifestation can sometimes be challenging to differentiate from other skin diseases, like eczemas. Superficial fungal infections are treated with oral and/or topical antifungal therapy; the treatment strategy depends on the severity of infection, anatomical location, and detected pathogen (6, 7).

Recent publications suggest that the overall prevalence of superficial fungal infections has increased over the years, but published data on children’s infections is limited, especially in Europe and Finland (8–11). The aim of this study is to investigate the epidemiological factors and treatment patterns of superficial fungal infections in children in a hospital providing secondary care for 410,000 inhabitants of Northern Finland between the years 2000 and 2021.

## MATERIALS AND METHODS

The study population included all patients aged under 18 years who visited the dermatology outpatient clinic of Oulu University Hospital (OUH) between 2000 and 2021 and who received a diagnosis code for fungal infection. After searching electronic health records (EHR) for the following International Classification of Diseases (ICD)−10 codes: B35.0 (tinea capitis), B35.1 (tinea unguium of toes or fingers), B35.3 (tinea pedis), B35.4 (tinea corporis), and B35.6 (tinea cruris), patients’ EHR data were reviewed and collected manually by KK. Patients with a final diagnosis of superficial fungal infection confirmed with either NAAT, fungal culture, or skin biopsy was included. Additionally, those cases whose diagnosis of superficial tinea was clinically diagnosed by a dermatologist and for whom treatment for tinea was initiated were retrospectively reviewed by dermatologists SPS and LH based on clinical photos and patient history and were included in the study if the case description also corresponded to that of superficial tinea after this retrospective review. Cases with superficial yeast infections (e.g., *Malassezia* and *Candida* species) were excluded. During the study period, fungal cultures were performed in accredited clinical mycology laboratories in Finland, and NAAT was performed with DermaGenius 2.0 complete multiplex kit (PathoNostics).

Data collection was carried out using the electronic REDCap (Research Electronic Data Capture) system, and information about preselected items (Table 1) was collected. If ethnicity was not mentioned in the EHR, in these cases, it was classified to be Finnish based on the patient information (i.e., more detailed patient history and name information).

**TABLE 1:** Characteristics of the study population

Total, n	94
Ethnicity, n (%)	
Native Finn (both parents)	77 (82.8%)
One parent is foreign	1 (1.1%)
Both parents are foreign	15 (16.1%)
Sex, n (%)	
Female	46 (48.9%)
Male	48 (51.1%)
Age, range	1-17
Mean (MD)	9.0 (4.4)
Median (Q1,Q3)	9.0 (6.0, 13.0)
The duration of symptoms before the referral to dermatology clinic	
<1 month	5 (5.4%)
1–< 3 months	10 (10.8%)
3–6 months	16 (17.2%)
Over 6 months	34 (36.6%)
Information not available	28 (30.1%)
Referral from	
Primary health care	52 (55.3%)
Pediatric department	16 (17.0%)
Private practitioner	12 (12.8%)
Other^*a*^	14 (14.9%)
Pre-selected comorbidities^*b*^	
Type 1 diabetes	5 (5.3 %)
Immunosuppressive medication or condition	3 (3.2%)

^*a*^ i.e., tinea diagnosed in a sibling of an index case or tinea as a secondary diagnosis in a follow-up visit of another disease (like atopic dermatitis or psoriasis), or no mention of the referral unit found in the electronic health records (EHR).

^*b*^ Type 1 diabetes, type 2 diabetes, previous organ transplant, immunosuppressive medication or immunosuppressive condition, and obesity were searched in the EHR.

### Statistical analyses

Data are presented as means, standard deviation and range, and as proportions for categorical variables. A Pearson’s Chi‐squared test and Fisher’s exact test, when appropriate, were used to test differences between variables. The statistical analyses were conducted using the R software package version 4.0.2 (https://cran.rstudio.com) and *P* < 0.05 was considered statistically significant.

## RESULTS

**Fig 1:**
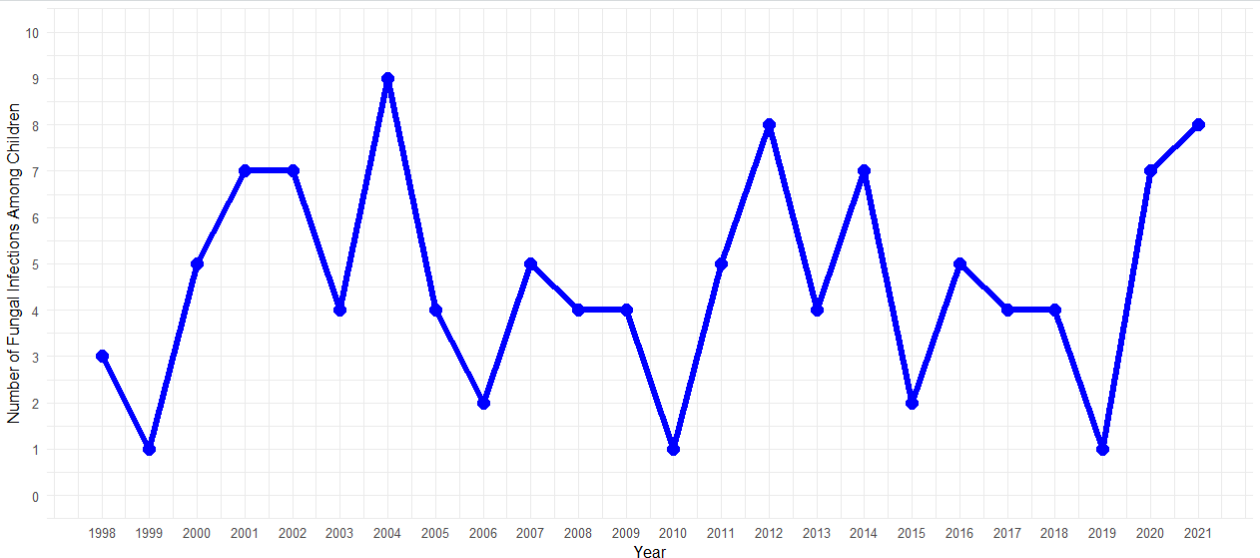
Cases of superficial fungal infections in children and adolescents diagnosed in the dermatology clinic of Oulu University Hospital during 2000–2021.

The data search returned 109 cases with the aforementioned diagnoses. Of these, 94 cases with 111 fungal infections fulfilled the inclusion criteria. During the study period, the overall number of cases with superficial fungal infection stayed rather stable, and the annual number of diagnoses ranged from 1 to 7 cases. (Fig. 1)

There were slightly more males (*n* = 48, 51.1%) than females (*n* = 46, 48.9%) in the study population. The mean age of the patient was 9.1 years. The majority of the study population was ethnically Finnish (*n* = 77, 82.8%), ie., both of their parents were Finnish. (Table 1). Type 1 diabetes (*n* = 5, 5.3%) and immunosuppressive medication or condition (*n* = 3, 3.2%) affected a minority of the cases, and there were no cases with history of organ transplantation. The most common suspected sources of infections were family members, followed by pets.

The most common fungal infection was tinea unguium of toenails (*n* = 42, 44.7%), followed by tinea capitis (*n* = 24, 25.5%) and tinea pedis (*n* = 22, 23.4%). (Table 2). The most common causative pathogen overall was *T. rubrum* (*n* = 53, 56.4%), which was also the most common pathogen found in cases of tinea unguium (*n* = 38/41, 92.6%) and in tinea pedis (*n* = 12, 54.5%). Tinea capitis was most frequently caused by *T. violaceum* (*n* = 9, 37.5%).

**TABLE 2:** Characteristics of superficial fungal infections by anatomical sites^*h*^^,^^*i*^

	Toe nail	Fingernail	Tinea pedis	Tinea corporis	Tinea capitis	Tinea cruris
**N, %**	42	3	22	19	24	1
Detected pathogen						
						
*T. interdigitale*	0	0	1 (4.6)	0	0	0
*T. rubrum*	36 (83.7)	2 (66.7)	12 (54.5)	3 (15.8)	0	0
*T. tonsurans*	0	0	0	2 (10.5)	1 (4.2)	0
*T. mentagrophytes*	1 (2.3)	0	1 (4.6)	3 (15.8)	3 (12.5)	0
*T. violaceum*	0	0	0	2 (10.5)	9 (37.5)	0
*T. benhamiae*	0	0	0	1 (5.3)	0	0
*T. verrucosum*	0	0	0	0	0	0
*M. canis/M. ferrugineum*	0	0	0	0	3 (12.5)	0
*M. audouinii*	0	0	0	0	2 (8.3)	0
*E. floccosum*	0	0	0	0	0	0
Other^*a*^	0	1 (33.3)	0	0	2 (8.3)	0
Diagnostic method^*b*^						
Fungal culture	30 (71.4)	2 (66.7)	10 (45.5)	11 (57.8)	20 (83.3)	0
Nucleic acid amplification method^*c*^	8 (19.0)	1 (33.3)	4 (18.2)	3 (15.8)	4 (16.7)	0
Clinical presentation^*d*^	4 (9.52)	0	11 (50.0)	7 (36.8)	3 (12.5)	1 (100)
Skin biopsy	0	0	0	1 (5.26)	1 (4.2)	0
Fungal infection as a reason for referral	34 (80.1)	1 (33.3)	9 (40.1)	10 (52.6)	15 (62.5)	0
Suspected source of infection						
Family member	8 (20.5)	0	1 (4.5)	3 (15.8)	2 (8.3)	0
Hobby	0	0	0	0	4 (16.7)	0
Pet	0	0	1 (4.5)	6 (31.6)	3 (12.5)	0
Friend/school	0	0	0	1 (5.2)	1 (4.2)	0
Traveling abroad	0	0	0	1 (5.2)	2 (8.3)	0
Unknown	34 (80.1)	3 (100)	20 (90.1)	8 (42.1)	12 (50.0)	1 (100)
Treatments used						
Topical antifungal only	8 (18.6)	1 (33.3)	13 (59.1)	7 (36.8)	2 (8.33)	1 (100)
Oral antifungal only	28 (67.7)	2 (66.7)	4 (18.2)	6 (31.6)	7 (29.2)	0
Combination of topical and oral antifungals	5 (11.9)	0	5 (22.7)	6 (31.6)	15 (62.5)	0
No treatment^*e*^	1 (2.4)	0	0	0	0	0
Treatment response evaluated	20 (47.6)	2 (66.7)	14 (66.7)	15 (78.9)	21 (87.5)	1 (100)
Outcome of the infection^*f*^						
Completely resolved	3 (15.0)	1 (50.0)	12 (85.7)	11 (73.3)	10 (50.0)	1 (100)
Partially resolved	16 (80.0)	1 (50.0)	2 (14.3)	4 (26.7)	8 (40.0)	0
Not resolved^*g*^	1 (5.0)	0	0	0	2 (10.0)	0

^*a*^ *T. terrestre*, *T. equinum*.

^*b*^ Multiple methods were used in some cases.

^*c*^ Used in OUH since 2015.

^*d*^ Also included are cases in which a fungal sample had been taken but the exact pathogen could not be retrospectively defined.

^*e*^ Patient/caregiver refused treatment.

^*f*^ *N* = 64; Based on the responsible clinician’s expert opinion in the follow-up visit.

^*g*^ Recovery was not seen or patient was lost from follow-up.

^*h*^ Due to the retrospective design, some missing data exist.

^*i*^ *T: Trichophyton; E: Epidermophyton; M: Microsporum*.

Over half of the cases were referred to a dermatologist’s evaluation from primary health care (55.3%). A superficial fungal infection or a suspicion of one was the reason for referral in 73.4% of cases, and the diagnosis of superficial tinea was most commonly set before the dermatologist’s evaluation in tinea unguium of the toes (80.1%) and in tinea capitis (62.5%). The identification of tinea unguium of the toes was statistically significantly better than in tinea corporis (*P* < 0.05) or tinea pedis (*P* < 0.5). Among those (*n* = 32, 33.7%) sent for referral without a suspicion of tinea, the time from symptom onset to diagnosis was less commonly ≥3 months compared with those referred with a suspicion of tinea (38.7% vs 60.3%), but due to the low number of cases, this did not reach statistical significance.

Oral or combination treatments were most commonly used for tinea capitis, as almost all (91.7%) of those cases were treated with either oral or combination treatments. Topical treatment was used as the only treatment in over a third of the tinea corporis cases. Overall, the treatment response was evaluated in 66.1% of cases during their clinical care, and complete or partial resolution was observed in 94.6% of them.

## DISCUSSION

In our study, 95 children were diagnosed with superficial fungal infection in secondary care during the 21-year study period, strengthening the previous understanding that fungal infections are rather rare in children. The most common diagnoses were tinea unguium, tinea capitis, and tinea pedis, of which tinea capitis is rarely seen in adults (12, 13).

The number of cases in our study was relatively low when compared with other studies performed in pediatric populations in secondary care. In an Indian study conducted in 2021–2022, there were 306 confirmed cases of tinea caused by dermatophytes in patients younger than 15 years old (14). A Polish study reported *n* = 165 children with fungal infection during the 5-year study period (15). The differences can most likely be explained by different geographical locations and socioeconomic conditions, e.g., the warmer climate in India may promote the growth of dermatophytes (1). It is also possible that treatment practices and the practices of referring children to secondary care vary between countries. Moreover, we excluded the cases with yeasts and molds, which differ from the Polish study.

We speculate that the small number of cases in the present study may be partly due to poor recognition of superficial tinea infections in children, as a third of the diagnoses were set for cases referred with a different (most commonly “dermatitis”) diagnosis. In addition, there seemed to be a delay between symptom onset and treatment initiation, with over half of the patients experiencing symptoms for over 6 months before getting a treatment plan in the dermatology clinic. However, those not suspected of having a superficial fungal infection had a shorter delay. Unfortunately, the actual time of symptom onset was often missing in the EHR and thus we were unable to further analyze these differences. In general, fungal infections may be challenging to diagnose and to differentiate from other skin diseases, leading to empirical or incorrect treatment before referral and thus resulting in diagnostic/treatment delays. Similar findings with a remarkable diagnostic delay have been previously described in the adult population, in which the diagnostic delay was over months in 46% of cases (12).

Our results demonstrate quite a stable number of superficial fungal infections during the study period of over two decades. In contrast, other studies in children have found an increasing trend (14, 16), but no studies have been conducted in or near our geographical location. However, a study in which data were collected from various European countries has shown increasing cases of tinea capitis (17). However, there is a lack of studies that have comprehensively considered the different types of tinea. Nevertheless, there are studies with similar findings to ours from outside Europe; a follow-up study from between 1996 and 2005 from Brazil showed no increase in the number of cases in children (18). The increase in travel and immigration would have been expected to lead to a rise in the number of cases in children, as has been shown to happen in adults (12).

In our study, the most prevalent causative agent was *T. rubrum* (56.4%), which primarily caused tinea unguium and tinea pedis in children and adolescents. This aligns with our previous findings among adults (12) and with a Polish study in which the most common causative of tinea unguium was *T. rubrum* (15). Correspondingly, in a previous Finnish study during 1982–1990, the most common species among both adults and children was *T. rubrum* (19). Additionally, *T. violaceum* (13.6%) and *T. mentagrophytes* (9.9%) were the second and third most common causatives, mostly causing tinea capitis and tinea corporis. Also, *T. tonsurans* and *T. mentagrophytes* were frequently found to cause tinea corporis. Our findings align with previous studies showing that *T. rubrum* is still the predominant pathogen worldwide (11). However, the prevalence of other pathogens varies across studies, influenced by the differences in common types of tinea and geographic factors. For example, tinea capitis shows more variability, with *M. canis, T. violaceum*, and *T. tonsurans* commonly reported as causative pathogens (1). However, the small number of cases in our study may limit comparisons regarding pathogen prevalence. Globally, the most common causative of tinea capitis is *M. canis*. Antifungal-resistant *T. indotineae* (*T. mentagrophytes* genotype VIII) has been reported in India and is increasingly being detected in Europe (20). Cases have also been reported in children in the United Kingdom (21). Antifungal resistance has also been observed in cases of tinea capitis in a Pakistan study in 2019 (22). According to the Pakistani study, resistance occurs among various dermatophytes.

In our study, most cases of tinea pedis were treated with topical treatments, while other types of tinea were predominantly treated with systemic or combination therapies. According to Finnish treatment guidelines (4), tinea pedis can be treated using either method.

A strength of our study is its retrospective design with a long, two-decade follow-up period. All patients were diagnosed by experienced dermatologists, and the vast majority of superficial fungal infections were verified by fungal culture or NAAT. We admit that we also included some cases that were diagnosed by clinical picture since, for example, it is acceptable to treat fungal infection in toe webs without fungal sampling according to the Finnish Current Care Guidelines (4). Although there are no pediatric dermatologists in Finland, and thus, it is recommended to refer children with tinea to dermatology clinics, it is possible that our data are missing some cases treated solely in pediatric secondary care. Furthermore, as this was a retrospective study, some data were not available in the EHR, which may have caused some bias. Since the study population included only children from an area with a mainly Caucasian population, the study results may not be generalized to other populations. Moreover, as this study was performed in secondary care, the results are comparable only with studies with a similar study design. Thus, we are unable to estimate the overall incidence of superficial fungal infections in our area during the study time.

In conclusion, this study demonstrates that superficial fungal infections are rather uncommon in the Finnish pediatric population and, unfortunately, they may be poorly recognized. Awareness of the clinical presentation of fungal infections should be increased among physicians to better differentiate them from other dermatological conditions and thus prevent diagnostic delays and spreading of infections. Furthermore, although we did not demonstrate an increasing trend in epidemiology during the past 20 years, physicians must be alert for possible changes in incidence in the future, i.e., due to increasing travel of families and the emergence of antifungal-resistant species.
